# Seroprevalence of Alphaviruses (*Togaviridae*) among Urban Population in Nouakchott, Mauritania, West Africa

**DOI:** 10.3390/v15071588

**Published:** 2023-07-20

**Authors:** Bedia Abdoullah, Guillaume André Durand, Leonardo K. Basco, Ahmed El Bara, Mohamed Abdallahi Bollahi, Laurent Bosio, Manon Geulen, Sébastien Briolant, Ali Ould Mohamed Salem Boukhary

**Affiliations:** 1 Unité de Recherche Génomes et Milieux (GEMI), Université de Nouakchott, Nouveau Campus Universitaire, Nouakchott BP 5026, Mauritania; bediaabdoullah@gmail.com; 2 National Reference Center for Arboviruses, National Institute of Health and Medical Research (Inserm) and French Armed Forces Biomedical Research Institute (IRBA), 13005 Marseille, France; guillaume.durand@inserm.fr (G.A.D.); laurent.bosio@inserm.fr (L.B.); manon.geulen@inserm.fr (M.G.); 3 Unité des Virus Émergents (UVE: Aix-Marseille Univ-IRD 190-Inserm 1207), 13005 Marseille, France; 4 Aix Marseille Univ, IRD, AP-HM, SSA, VITROME, 13005 Marseille, France; lkbasco@yahoo.fr (L.K.B.); sbriolant@wanadoo.fr (S.B.); 5 IHU-Méditerranée Infection, 13005 Marseille, France; 6 Institut National de Recherche en Santé Publique, Nouakchott BP 695, Mauritania; elbaraahmed@yahoo.fr (A.E.B.); bollahi@yahoo.com (M.A.B.); 7 Unité de Parasitologie Entomologie, Département de Microbiologie et Maladies Infectieuses, Institut de Recherche Biomédicale des Armées (IRBA), 13005 Marseille, France

**Keywords:** arbovirus, alphavirus, chikungunya, O’nyong-nyong, Semliki Forest, seroprevalence, ELISA, neutralization, Nouakchott, Mauritania

## Abstract

The presence of alphaviruses, such as chikungunya virus (CHIKV), has never been reported in Mauritania. We assessed the seroprevalence of CHIKV among Nouakchott residents. A cross-sectional study involving 1300 non-febrile patients consulting at the Nouakchott hospital center was conducted between January and June 2021. The presence of anti-CHIKV IgG and neutralizing antibodies against CHIKV, O’nyong-nyong virus (ONNV), and Semliki Forest virus (SFV) was determined by an enzyme-linked immunosorbent assay (ELISA) and a serum neutralization test, respectively, and the associated risk factors were investigated. Of the 1300 study participants, serological evidence of previous exposure to CHIKV was observed in 37 individuals (2.8%). Sex, age, reported use of repellants, and bed net ownership and usage were not associated with CHIKV seropositivity. Our results showed the co-circulation of two other alphaviruses, ONNV and SFV, in Nouakchott in 30 (2.3%) individuals. This is the first study that documents the co-circulation of CHIKV, ONNV, and SFV in Mauritania, albeit at low prevalence. Surveillance and routine testing for alphaviruses and other arboviruses in symptomatic patients should be implemented in health facilities to assess the health burden associated with these viruses. Efforts should also be made to strengthen the vector control measures.

## 1. Introduction

The genus *Alphavirus* (family *Togaviridae*) includes medically important and emergent viruses that cause major epidemics in Africa, Europe, Oceania, South America, and Southeast Asia [1,2]. Among 40 known types and subtypes of alphaviruses, chikungunya virus (CHIKV) and O’nyong-nyong virus (ONNV) are currently the most important members associated with diseases in humans in Africa [3,4,5]. CHIKV and ONNV are closely related single-stranded RNA viruses within the Semliki Forest antigenic complex [6,7]. Many alphaviruses are maintained in nature through mosquito–vertebrate–mosquito transmission cycles occurring in both sylvatic and urban environments [8,9]. These cycles are maintained by a diversity of mosquito species. Transmission of CHIKV and Semliki Forest virus (SFV) to humans involves *Aedes* (Stegomyia) *aegypti* and *Ae.* (Steg.) *albopictus* mosquitoes [10,11]. Other *Aedes* spp., such as *Ae. furcifer* and *Ae. africanus*, may be involved to a lesser degree in the transmission of CHIKV in sylvatic cycles [9,12]. *Anopheles funestus* and *An. gambiae* are the main vectors of ONNV [13].

Clinically, human illness due to alphaviruses is often self-limited [14]. The main clinical symptoms of Old World alphaviruses include fever, arthralgia, skin rash, headache, and malaise. Other symptoms, such as myalgia and retro-orbital pain, may also be observed in some patients [15,16]. In 2023 (as of 9 March), 114,181 cases and 43 deaths attributed to CHIKV disease have been reported globally, mostly in Paraguay [17]. The currently available epidemiological data suggest that ONNV occurs exclusively in Africa [3]. Mortality due to ONNV infection has not been reported. The morbidity caused by ONNV is not well-known due to the absence of surveillance in Africa.

The clinical manifestations of Old World alphavirus infections are closely similar to those caused by other arboviruses, such as dengue virus (DENV), except for arthralgia that can persist for several years in 5% of patients with CHIKV infection [18]. In countries where DENV is endemic, as in most West African countries including Mauritania, infections due to alphaviruses, in particular CHIKV and ONNV, are probably misdiagnosed and/or underreported [13]. In West Africa, autochthonous cases of CHIKV have been reported in Nigeria, Burkina Faso, Senegal, Benin, Ghana, Guinea, and Côte d’Ivoire [19,20]. In Senegal, a country bordering Mauritania, CHIKV epidemics have been reported since 1960, with recurrent, limited outbreaks up to 2010 [8,11,21,22]. An ONNV epidemic in Senegal and many other countries in East and West Africa occurred in the early 1960s [13]. As for Mali, another sub-Saharan West African country sharing a common border with Mauritania, there is some relatively recent (i.e., 2009–2016) serological evidence of a previous exposure of the local human populations to CHIKV and ONNV [4,23].

Infections due to alphaviruses have not yet been documented in Mauritania. However, dengue fever epidemics have occurred repeatedly in Nouakchott since 2014. Dengue virus is a single-stranded RNA virus that belongs to the genus *Flavivirus*. Like alphaviruses, DENV is an arthropod-borne virus (i.e., arbovirus). Dengue virus isolates implicated in the 2014 epidemic in Nouakchott were initially detected by a rapid diagnostic test for dengue, and their protein envelope sequences were further characterized [24]. Since DENV and CHIKV are both transmitted by the same vector, *Ae. aegypti*, known to be present in abundance in Nouakchott [25,26], and the presence of CHIKV is highly probable or confirmed in the neighboring sub-Saharan countries, we hypothesized that there is a high risk of possible CHIKV transmission in Nouakchott. Moreover, cases of CHIKV and DENV co-infection have been reported elsewhere [27,28]. Furthermore, according to the National Institute of Public Health Research (INRSP) of the Mauritanian Ministry of Health, only 10% of the suspected “dengue-like” clinical cases screened at the laboratory were due to DENV, leaving 90% of febrile, symptomatic cases without any etiologic diagnosis. It is within this context that we set the objective of the present serology-based survey to determine the previous exposure of non-febrile patients to some commonly occurring alphaviruses in the urban population in Nouakchott.

## 2. Materials and Methods

### 2.1. Study Site

**Figure 1:**
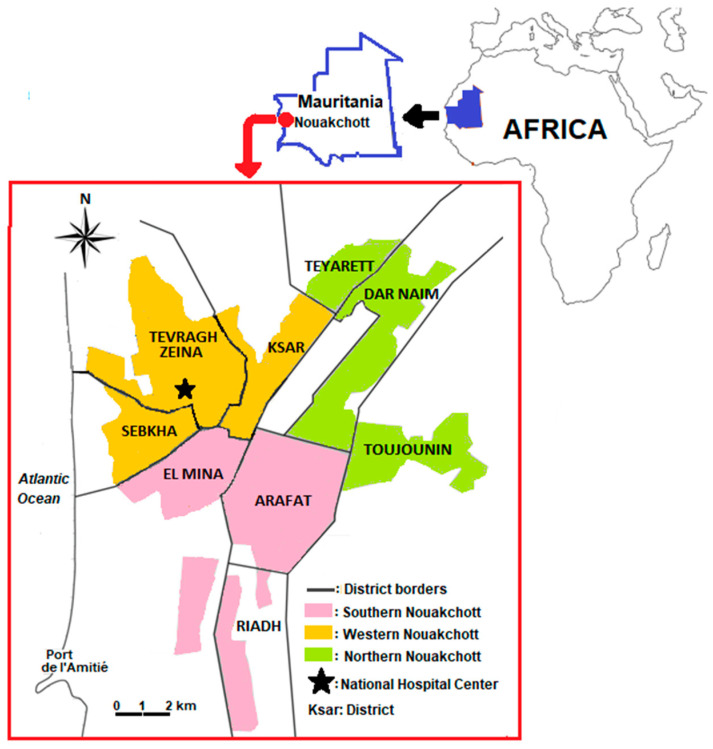
Map of Nouakchott, Mauritania, showing the location of the study site: the National Hospital Center located in the Tevragh Zeina district. Colored areas correspond to three Nouakchott regions: northern Nouakchott region (light green), including the districts of Dar Naim, Teyarett, and Toujounin; southern Nouakchott region (salmon pink) with the districts of Arafat, Riadh, and El Mina; and western Nouakchott region (orange) corresponding to the districts of Sebkha, Tevragh Zeina, and Ksar. The large white band in a rectangular shape in the Dar Naim district represents the former international airport (closed since 2016 due to the opening of the new international airport situated outside the city). The Atlantic Ocean lies to the west of the city. Adapted from [32]. Figure available online: http://books.openedition.org/pupo/docannexe/image/17487/img-2.jpg (accessed on 15 September 2022).

The study was conducted in Nouakchott, the capital city of Mauritania, situated in the Atlantic coastal zone (Figure 1). In 2015, Nouakchott was administratively divided into three regions locally called “Wilayas,” each consisting of three of nine districts that formerly composed Nouakchott. The northern Nouakchott region or “Wilaya” includes the districts of Teyarett, Dar Naim, and Toujounin; the southern Nouakchott region or “Wilaya” comprises the districts of El Mina, Riadh, and Arafat; and the western Nouakchott region or “Wilaya” is composed of the Tevragh Zeina, Ksar, and Sebkha districts. The city is densely populated with approximately 1,150,000 inhabitants distributed across a surface area of 1000 km^2^, corresponding to a population density of 1150 inhabitants per km^2^, 47.8% of whom are women, and 60.7% are between 15 and 60 years of age [29]. The climate in Nouakchott is characterized as Saharan, i.e., low annual rainfall (<100 mm on average) and a mean annual temperature and humidity of 27 °C and 56.5%, respectively. Despite the Saharan climate, urbanization, in particular the development of a potable water distribution system, has created an environment that is highly conducive to the creation of mosquito larval habitats [30,31]. Various mosquito species, including *Anopheles* spp., *Aedes* spp., and *Culex* spp., abound in the city [26,30].

### 2.2. Study Design and Sample Collection

A cross-sectional serological screening was conducted during the period from January to June 2021 in the Nouakchott national hospital center (NNHC), situated in the city center. Of the six existing hospital centers in Nouakchott, the NNHC is the oldest hospital which receives the highest number of patients from all nine districts of Nouakchott. As the purpose of this study was to estimate the prevalence of anti-CHIKV immunoglobulin G (IgG), the included participants were non-febrile (axillary temperature < 37.5 °C), aged > 1 year, and visiting the NNHC laboratory for a routine medical checkup. The minimum sample size (*n*) of participants to be included was 385, estimated according to the following formula:

$$n=\frac{\delta^{2}\times \;P\left(1-P\right)}{M^{2}}$$

where δ is the standard deviation set at 1.96, corresponding to a 95% confidence interval (95% CI), P is the expected prevalence assumed as 50%, and M is the margin of error set at 5%.

After informed consent was obtained from the participants (or their legally authorized guardians), serum samples were collected. Approximately 3 mL of blood was obtained, by venipuncture into a sterile blood collection tube without anticoagulant, from each participant. The serum was separated by centrifugation and stored at −20 °C until it was tested for anti-CHIKV IgG. A questionnaire, including socio-demographic characteristics (age, sex, their district of residence), information on bed net ownership and utilization, and use of mosquito repellants, was administered to each participant (or their legal guardians).

### 2.3. IgG Antibody Testing

The samples were tested for anti-CHIKV IgG antibodies using an in-house enzyme linked immuno-sorbent assay (ELISA). Briefly, the plates were coated with an inactivated virus supernatant from the French Centre National de Référence (Marseille, France). The fixation of anti-CHIKV IgG was performed with a goat anti-human IgG conjugate labeled with Fcy fragment-specific affinity-purified horseradish peroxidase (Jackson ImmunoResearch Europe Ltd.; Ely, Cambridgeshire, UK). The absorbance was read by a microplate ELISA reader (Bio-Rad Benchmark™ microplate reader; Bio-Rad, Marnes-la-Coquette, France) at 450 nm. We interpreted the ELISA assay for CHIKV as follows: (1) sample absorbance/negative control absorbance ≤ 3, negative (values of sample absorbance/negative absorbance between 2.5 and 3, usually considered as undetermined, were considered as negative in this study in order to limit the risk of false positives); (2) sample absorbance/negative control absorbance > 3, positive.

### 2.4. Serum Neutralization Assay

A microneutralization assay was performed to detect neutralizing antibodies in positive samples for anti-CHIKV IgG. After filtration through a 0.22 µm filter, 100 µL of test sera were diluted in phosphate buffered saline (PBS), and a two-fold serial dilution from 1:20 to 1:360 was prepared in a 96-well plate. For each dilution, contact was performed with a 50 median tissue culture infectious dose (TCID50) for one hour at 37 °C in a 5% CO_2_ incubator. The viral strains used were CHIKV (La Reunion 2005), ONNV (Chad 2004), and SFV (Africa 1997). The complexes were then added to Vero cells (American Tissue Culture Collection (ATCC) CCL-81, 1.3 × 10^5^ cells/well). After four days of incubation, the cytopathogenic effects were investigated with a light microscope by a trained operator under biosafety level 3 (BSL-3) conditions.

The neutralizing titer was defined as the inverse of the highest dilution resulting in an infectious reduction of 50%. A titer lower than 1:20 was considered as negative if cytopathic effects were not observed. Samples were considered as positive if the titer was >1:20, i.e., positive for ONNV if ONNV titers were at least two-fold higher than CHIKV titers, and CHIKV positive if CHIKV titers were at least four-fold higher than ONNV titers [33]. The threshold was lower for ONNV than CHIKV because of the unique one-way cross-reactivity between CHIKV and ONNV, i.e., CHIKV antibodies are more likely to cross-react with ONNV antigens than ONNV antibodies with CHIKV antigens [4]. All other situations were interpreted as ‘equivocal.’ The SFV results were interpreted independently, as follows: titer <1:20 was interpreted as negative; and all other titers ≥1:20 were interpreted as positive.

### 2.5. Statistical Analysis

The ELISA results and data collected from the questionnaires were entered into an Excel datasheet (Microsoft Inc., Redmond, WA, USA) and analyzed using R software (v4.2.0; R Foundation for Statistical Computing, Vienna, Austria; https://www.r-project.org/, accessed on 15 September 2022). We tested for associations between age, sex, bed net and repellant uses, distribution of residence, and CHIKV seropositivity using logistic regression (LR), with the level of significance set at <0.05 and the crude odds ratio (cOR) set at a confidence interval (CI) of 95%.

## 3. Results

### 3.1. Seroprevalence of Anti-CHIKV IgG

A total of 1300 participants from both sexes were included in the present study of whom 46.8% (601/1300) were males and 53.1% (691/1300) were females, resulting in a male-to-female sex ratio of 0.87 (Table 1). Of the 1300 serum samples tested for anti-CHIKV IgG antibodies by ELISA, 37 (2.8%) were positive (1% of the samples, *n* = 14, fell within a 10% range of the assigned cut-off value (2.7–3.0)). Seropositivity was more frequent among males (3.1%) than in females (2.6%), but the difference was not statistically significant (cOR = 1.2, 95%CI (0.6–2.3), *p* = 0.58, LR).

The participants’ ages ranged from 1 to 95 years, with a mean and median age of 39.7 and 38.0 years, respectively (Table 1). Anti-CHIKV IgG was found in all age groups, but mostly in the age group ≥50 years old, in whom a seroprevalence of 4.1% was observed (cOR = 2.0, 95%CI 0.6–6.9, *p* = 0.29, LR).

Participants were from all nine districts of Nouakchott, with the lowest number residing in the Ksar district (*n* = 73) and the highest number in the Arafat district (*n* = 241). Seropositive cases were found in all nine districts of Nouakchott (Table 2). When the study participants were grouped according to the new administrative division of Nouakchott, i.e., the three “Wilayas” within the city of Nouakchott, the differences in the seropositivity rates among these three “Wilayas” were not statistically significant (*p* > 0.05, LR). The highest rate of anti-CHIKV IgG positivity was observed among those residing in the southern Nouakchott region, which includes the districts of Arafat, El Mina, and Riadh, with a seroprevalence of 3.5%. By contrast, the lowest seroprevalence (2.2%) observed in the present study was among the participants residing in the northern Nouakchott region, comprising the Teyarett, Dar Naim, and Toujounin districts.

Repellent users had lower rates of seropositivity (1.3%) compared to non-users (3.1%), but the difference did not reach statistical significance (cOR = 0.4, 95%CI 0.1–1.3, *p* = 0.14, LR) (Table 1). Likewise, the comparison of seropositivity rates between bed net users and bed net non-users, and those who claimed to sleep often under a bed net and those who did not sleep under a bed net, did not show any statistically significant difference (cOR = 1.2, 95%CI 0.6–2.3, *p* = 0.64, LR and cOR = 0.8, 95%CI 0.4–1.4, *p* = 0.39, LR, respectively).

### 3.2. Serum Neutralization Assay

The results of the microneutralization assay are presented in Table 2. According to our test interpretation, 2 of 36 samples (5%) had neutralizing antibodies against CHIKV, 19 of 36 (52.7%) samples had neutralizing antibodies against ONNV, and 18 of 36 (50%) samples were shown to have neutralizing antibodies against SFV. The geometric mean titers (GMT) were 905 for CHIKV, 62 for ONNV, and 37 for SFV. Despite high titers, 11 samples (29.7%) were interpreted to be equivocal for CHIKV and ONNV.

## 4. Discussion

Mauritania is known to be the epicenter of various arboviruses in West Africa, particularly Rift Valley fever (Phlebovirus) and Crimean-Congo hemorrhagic fever (Orthonairovirus) viruses, which have caused recurrent epidemics during the last four decades [34,35,36,37,38,39,40,41,42]. In addition, annual epidemics of dengue fever have been documented in Nouakchott since 2014, following the introduction and establishment of the anthropophilic *Ae. aegypti* mosquito, which is known to be well adapted to urban settings [24,30].

The present seroprevalence survey reports for the first time on the circulation of three additional arboviruses of the genus *Alphavirus* in the human host, namely CHIKV (0.2%), ONNV (1.5%), and SFV (1.4%), 0.8% being equivocal for both CHIKV and ONNV, with an overall prevalence of anti-CHIKV IgG positivity of 2.8% among Nouakchott residents. A similar seroprevalence rate (2.7%) for anti-CHIKV-IgG was found in a survey carried out in 2014 in 1465 Senegalese nomadic pastoralists residing in the northeastern regions of Senegal [43]. In an earlier study (2009–2013) conducted in Mali, an IgG positivity rate of 6.6% was reported [23]. In a later (2016) survey conducted in southern Mali, a much higher anti-CHIK-IgG seroprevalence (13%), as well as that of ONNV (30%), was predicted [4]. However, that Malian study was based on statistical modeling. The actual finding from that study based on a serum neutralization assay was a seroprevalence of 1.8% for CHIKV and 26.9% for ONNV. The results of the studies based on similar serological tests and conducted in a West African sub-region, where the three countries share a common frontier, were therefore comparable in southern Mauritania (i.e., Nouakchott), northeastern Senegal, and southern Mali. Elsewhere in Africa, where serological surveillance has been performed in recent years, a slightly higher seroprevalence of 4.2% was found in Ghana [44], but the seroprevalence (anti-CHIKV IgG and/or IgM) was much higher in the Democratic Republic of Congo (30.6%) [45], Zambia (36.9%; 79/214) [46], and Nigeria (25.1–41.3%) [7,47,48]. In East Africa, the following rates of seroprevalence, based on IgG against CHIKV, were reported in recent studies: 5.2% in Kenya [49], 28.0% in Tanzania [50], and 5.3–43.6% depending on the study site in Ethiopia [51,52,53]. In a meta-analysis on serological surveillance of CHIKV in Africa between 2008 and 2017, it was reported that, based on 23 selected published studies, the overall IgG seroprevalence for CHIKV was 6.4% (95% confidence interval, 9.1–25.2%) [54]. Based on these data, it can be reasonably presumed that the relatively low seroprevalence rates found in Mauritania, Senegal, and Mali (i.e., 1.8–2.8%) may suggest that CHIKV circulation is a relatively recent event in this part of the sub-region of West Africa. As for ONNV epidemiology in Africa, the recent data are incomplete, and further efforts are required to develop reliable diagnostic tools for the specific identification of this virus [3,21].

In our study, a relatively higher rate of IgG positivity was observed in the age group ≥ 50 years, in agreement with previous reports among older patients in Nigeria [47]. Moreover, a serological study conducted in school children aged 3 to 17 years in southern Mali showed that only 6.2% of those tested had positive IgG responses to the recombinant CHIKV E1 antigen, but increasing anti-CHIKV IgG levels were noted with increasing age [55]. This can be most likely attributed to the fact that the risk of viral exposure increases with the age of each individual [43,45,47,48,56]. However, in India, CHIK seroprevalence in older participants was not significantly different from the other age groups, indicating an epidemic pattern of transmission [57]. Moreover, males were more exposed to alphavirus infections in the present study, despite the recruitment of more female participants (sex ratio of 0.87). Other authors have also reported that males tend to be more often infected with an *Alphavirus* than females [47], while others did not observe any difference [43]. This sex bias in some studies may be attributed to cultural habits and behaviors, which predispose males to more bites by *Aedes* mosquitoes.

Patients with IgG antibodies to CHIKV, ONNV, and SFV were distributed in all Nouakchott districts, but a higher prevalence was observed among those residing in the southern districts of Arafat, Riadh, and El Mina. As with dengue, CHIKV, ONNV, and SFV are most often transmitted by *Ae. aegypti*, *An. gambiae*, and *An. funestus* mosquitoes. *Aedes aegypti* has become well adapted to the urban environment, where the presence of water storage containers, discarded water holding containers, and other debris in which stagnant water can accumulate ensures the creation of larval habitats [30,58]. Likewise, *Anopheles* spp., in particular *An. arabiensis*, have come to thrive in similar environmental conditions in Nouakchott, characterized by the presence of unpolluted, potable water supply distribution [59,60,61].

Neither the use of repellants nor bed net ownership and its utilization showed a protective effect against infection with an *Alphavirus* in the present study. Our finding is in agreement with that of other authors [43]. The majority of alphavirus infections detected in Nouakchott were ONNV which, unlike CHIKV and SFV, is transmitted by night-feeding *Anopheles* spp., as also reported in a Kenyan study [33]. To explain the higher seroprevalence of CHIKV in the southern districts of Nouakchott, a comparative entomological survey is needed to investigate the mosquito population density in different parts of the city. Further, serosurveillance would also be required to confirm the trend observed in the present study.

The interpretation of a seroneutralization assay for alphaviruses is challenging due to cross-reactivity between CHIKV and ONNV [21]. In our results, 100% of the samples with neutralizing antibodies against CHIKV also displayed neutralizing antibodies against ONNV, which implies full cross-neutralization. However, only 60% of the samples with neutralizing antibodies against ONNV displayed neutralizing antibodies against CHIKV. This asymmetrical cross-neutralization has also been reported by other authors [4]. Despite a high proportion of samples with equivocal test results (29.7%), our strategy of using at least two-fold dilution decreased the risk of misinterpretation. Moreover, 12 samples were only ONNV positive, with a titer up to 1:160 (GMT 42). In our strategy, we tested only the samples that tested positive for anti-CHIKV IgG with ELISA, that allowed us to find all CHIKV cases, but some ONNV cases could have been missed. However, there is currently no universally accepted titer threshold for positivity, and the differentiation between CHIKV and ONNV based on a two-fold titer difference is arbitrary [4,21]. Our data suggest that ONNV circulates in Mauritania, probably much more than CHIKV. In a recent work performed in southern Mali, a neighboring country of Mauritania, the authors came to a similar conclusion [4]. The search for neutralizing antibodies against SFV was also performed in the present study. Although SFV was first isolated in Uganda in 1942, epidemiological data on SFV in Africa are scarce, and recent data are lacking. One study related to an SFV outbreak in central Africa in 1987 reported the presence of arthralgia in all the infected patients, making SFV infection a possible differential diagnosis for CHIKV and ONNV infections [62]. Cross-neutralization with other alphaviruses is unknown, but the fact that CHIKV, ONNV, and SFV belong to the same serocomplex probably leads to cross-reactivity. In our results, the GMT of neutralizing antibodies against SFV was relatively low, but four samples displayed neutralizing antibodies only against SFV, suggesting that this *Alphavirus* is probably circulating in Mauritania at a low level. More investigations are needed to clarify and update the epidemiological situation of SFV in Africa.

Our study has some limitations. First, the presence of IgM in the collected sera was not screened to evaluate possible ongoing infections. Second, entomological investigations were not carried out during the study period to capture vector specimens for viral testing. Therefore, it was not possible to associate clinical data with vector abundance or mosquito infection rates. Third, the travel history of the participants was not recorded, precluding us from ascertaining whether the seropositive results were due to an autochthonous infection or travel-acquired infections from other CHIKV endemic areas. Lastly, as already mentioned above, cross-reactivity between CHIKV and ONNV is a technical problem that cannot be circumvented in serological studies.

## 5. Conclusions

Our data provides the first evidence on the circulation of alphavirus in Nouakchott. Since the clinical signs and symptoms of alphavirus infections are indistinguishable and persistent arthralgia can be very disabling, there is a need to implement systematic differential diagnosis at health facilities. Further, arbovirus surveillance at a national scale, as well as preventive measures, is warranted in Mauritania.

## Figures and Tables

**Table 1:** Association between the characteristics of the participants and anti-chikungunya virus IgG positivity.

Variable	No. Tested (%)	Anti-CHIKV IgG Positive *n* (%; 95% CI)	cOR (95% CI)	*p* Value
Overall	1300 (100.0)	37 (2.8; 2.0–3.9)	-	-
Sex Female Male	691 (53.1) 609 (46.8)	18 (2.6; 1.6–4.1) 19 (3.1; 1.9–4.8)	1 1.2 (0.6–2.3)	0.58
Age (yr) 0–<20 20–<35 35–<50 ≥50	141 (10.8) 448 (34.5) 345 (26.5) 366 (28.1)	3 (2.1; 0.4–6.1) 13 (2.9; 1.6–4.9) 6 (1.7; 0.6–3.8) 15 (4.1; 2.3–6.7)	1 1.4 (0.4–4.9) 1.2 (0.3–5.0) 2.0 (0.6–6.9)	0.62 0.77 0.29
Residence Northern Nouakchott (NN) Teyarett Dar Naim Toujounin Total in NN Western Nouakchott (WN) Sebkha Tevragh Zeina Ksar Total in WN Southern Nouakchott (SN) Riadh Arafat El Mina Total in SN	129 96 97 322 144 160 73 377 176 241 184 601	2 (1.6; 0.2–5.5) 3 (3.1; 0.7–8.9) 2 (2.1; 0.3–7.3) 7 (2.2; 2.0–7.5) 5 (3.5; 1.1–7.9) 2 (1.3; 0.2–4.4) 2 (2.7; 0.3–9.6) 9 (2.4; 0.7–8.9) 8 (4.5; 2.0–8.8) 10 (4.1; 2.0–7.5) 3 (1.6; 0.3–4.7) 21 (3.5; 0.3–4.7)	1 1.1 (0.4–3.0) 1.6 (0.7–3.9)	0.85 0.27
Use of impregnated bed net No Yes	1050 (80.7) 250 (19.2)	31 (3.0; 2.0–4.2) 5 (2.4; 0.9–5.2)	1 1.2 (0.6–2.3)	0.64
Sleeping under bed net Never Always	758 (58.3) 542 (41.7)	19 (2.5; 1.5–3.9) 18 (3.3; 2.0–5.2)	1 0.8 (0.4–1.4)	0.39
Repellent use Never Always	1073 (82.5) 227 (17.5)	34 (3.2; 2.2–4.4) 3 (1.3; 0.3–3.8)	1 0.4 (0.1–1.3)	0.14

Abbreviations: *n*: number of anti-CHIKV IgG positive samples; 95% CI: 95% confidence interval; cOR: crude odds ratio.

**Table 2:** Results of the microneutralization assay against the chikungunya, O’nyong-nyong, and Semliki Forest viruses.

Sample No.	District	CHIKV		ONNV		SFV	
		Titre	Interpretation	Titre	Interpretation	Titre	Interpretation
72	El Mina	<1:20	−	1:80	+	1:40	+
352	El Mina	1:640	+	1:160	−	1:20	+
1197	El Mina	<1:20	−	1:20	+	<1:20	−
212	Sebkha	<1:20	−	1:160	+	<1:20	−
232	Sebkha	<1:20	−	1:20	+	<1:20	−
235	Sebkha	1:20	−	1:80	+	1:40	+
1040	Sebkha	1:20	−	1:80	+	<1:20	−
1065	Sebkha	<1:20	−	1:20	+	<1:20	−
224	Riadh	1:160	Eq	1:160	Eq	<1:20	−
265	Riadh	1:20	−	1:160	+	<1:20	−
453	Riadh	1:1280	+	1:160	−	1:80	+
657	Riadh	1:80	Eq	1:160	Eq	1:40	+
690	Riadh	1:160	Eq	1:160	Eq	<1:20	−
759	Riadh	1:80	Eq	1:80	Eq	1:40	+
824	Riadh	1:40	−	1:160	+	<1:20	−
1111	Riadh	1:320	Eq	1:160	Eq	<1:20	−
252	Teyarett	<1:20	−	<1:20	−	1:20	+
834	Teyarett	1:40	Eq	1:80	Eq	<1:20	−
342	Arafat	1:20	−	1:160	+	1:20	+
380	Arafat	1:160	Eq	1:160	Eq	1:160	+
463	Arafat	<1:20	−	<1:20	−	1:80	+
599	Arafat	1:40	−	1:160	+	1:40	+
632	Arafat	<1:20	−	1:40	+	<1:20	−
780	Arafat	1:40	Eq	1:80	Eq	<1:20	−
1066	Arafat	<1:20	−	1:20	+	1:20	+
1214	Arafat	1:20	−	1:80	+	1:40	+
40,487	Arafat	1:80	Eq	1:160	Eq	1:20	+
584	Ksar	<1:20	−	<1:20	−	1:20	+
1295	Ksar	<1:20	−	<1:20	−	1:40	+
624	Tevragh Zeina	<1:20	−	1:160	+	1:20	+
873	Tevragh Zeina	1:160	Eq	1:160	Eq	1:80	+
703	Toujounin	1:80	Eq	1:160	Eq	<1:20	−
949	Toujounin	<1:20	−	1:40	+	<1:20	−
943	Dar Naim	<1:20	−	1:40	+	<1:20	−
1038	Dar Naim	<1:20	−	1:40	+	<1:20	−
1165	Dar Naim	<1:20	−	1:40	+	<1:20	−

Symbols and abbreviations: −: negative; +: positive; Eq: equivocal; CHIKV: chikungunya virus; ONNV: O’nyong-nyong virus; SFV: Semliki Forest virus.

## Data Availability

The data presented in this study are available on request from the corresponding author.
